# Trends in antibiotic resistance among bacteria isolated from blood cultures using a large private laboratory network data in India: 2008-2014

**DOI:** 10.1186/2047-2994-4-S1-O42

**Published:** 2015-06-16

**Authors:** S Gandra, N Mojica, A Ashok, BR Das, R Laxminarayan

**Affiliations:** 1 Center for Disease Dynamics, Economics & Policy, New Delhi, India; 2 Center for Disease Dynamics, Economics & Policy, Washington DC, USA; 3 SRL Diagnostics Ltd., Mumbai, India; 4 Public Health Foundation of India, Gurgaon, India; 5 Princeton Environmental Institute, Princeton, NJ, USA

## Introduction

Antimicrobial resistance surveillance is essential to track changes in microbial populations, estimate the magnitude of the problem and to design and evaluate interventions. However, there is no national level information on resistance among bacteria causing bloodstream infections in India.

## Objectives

The purpose of the study is to examine the extent and trends of antibiotic resistance among bacteria isolated in blood cultures from January 1, 2008 to December 31, 2014.

## Methods

Antibiotic susceptibility data were obtained from more than 275 microbiology laboratories spread across 24 states in India which are part of a large private laboratory network. We retrospectively examined trends of resistance in pathogenic bacteria (*Salmonella typhi/paratyphi*, *Escherichia coli*, *Klebsiellaspecies*, *Staphylococcus aureus*, *Acinetobacter species* and *Pseudomonas aeruginosa*) isolated from blood cultures for years 2008-2014.

## Results

A total of 136,915 unique blood cultures were obtained over the seven years period and total positive cultures were 17,494 (13%). The breakdown of the organisms by frequency of isolation include: coagulase negative staphylococcus (4488, 26%), *Salmonella sp*. ( *typhi/paratyphi*) (3202, 18%), *E.coli* (2191, 13%), *Klebsiella sp*. (1401, 8%), *S. aureus* (1053, 6%), *Acinetobacter sp*. (1021, 6%) *Pseudomonas sp*. (794, 5%) and others (3344, 19%). Ciprofloxacin resistance in *Salmonella sp*. increased from 13% in 2008 to 22% in 2014. Third generation cephalosporin resistance in *E.coli* was 74% in 2008 and increased to 80% in 2014 and in *Klebsiella sp*., it was 94% in 2008 and decreased to 80% in 2014. Carbapenem resistance in *Klebsiella sp*. was 22% in 2008 and increased to 60 % in 2014 and in *E.coli*, was 7% in 2008 and increased to 12% in 2014. Carbapenem resistance in *Acinetobacter sp*. was 73% in 2008 and decreased to 69% in 2014 and in *P. aeruginosa* it was 55% in 2008 and decreased to 37% in 2014. Methicillin resistance *S. aureus* increased from 50% in 2008 to 55% in 2014.

## Conclusion

Very high rates of resistance were observed to frontline and last-resort antibiotics among bacteria isolated from blood cultures in India.

## Disclosure of interest

None declared.

